# Monitoring and Prevalence Rates of Metabolic Syndrome in Military Veterans with Serious Mental Illness

**DOI:** 10.1371/journal.pone.0019298

**Published:** 2011-04-26

**Authors:** Sameed Ahmed M. Khatana, Joshua Kane, Tracey H. Taveira, Mark S. Bauer, Wen-Chih Wu

**Affiliations:** 1 Research Enhancement Award Program at the Providence Veterans Affairs Medical Center, Providence, Rhode Island, United States of America; 2 University of Pittsburgh School of Medicine, Pittsburgh, Pennsylvania, United States of America; 3 Butler Hospital, Providence, Rhode Island, United States of America; 4 University of Rhode Island College of Pharmacy, Kingston, Rhode Island, United States of America; 5 Department of Medicine at the Providence Veterans Affairs Medical Center, Warren Alpert Medical School of Brown University, Providence, Rhode Island, United States of America; 6 Department of Psychiatry, Harvard Medical School, Boston, Massachusetts, United States of America; 7 VA Boston Healthcare System, Boston, Massachusetts, United States of America; Leicester University, United Kingdom

## Abstract

**Background:**

Cardiovascular disease is the leading cause of mortality among patients with serious mental illness (SMI) and the prevalence of metabolic syndrome—a constellation of cardiovascular risk factors—is significantly higher in these patients than in the general population. Metabolic monitoring among patients using second generation antipsychotics (SGAs)—a risk factor for metabolic syndrome—has been shown to be inadequate despite the release of several guidelines. However, patients with SMI have several factors independent of medication use that predispose them to a higher prevalence of metabolic syndrome. Our study therefore examines monitoring and prevalence of metabolic syndrome in patients with SMI, including those not using SGAs.

**Methods and Findings:**

We retrospectively identified all patients treated at a Veterans Affairs Medical Center with diagnoses of schizophrenia, schizoaffective disorder or bipolar disorder during 2005–2006 and obtained demographic and clinical data. Incomplete monitoring of metabolic syndrome was defined as being unable to determine the status of at least one of the syndrome components. Of the 1,401 patients included (bipolar disorder: 822; schizophrenia: 222; and schizoaffective disorder: 357), 21.4% were incompletely monitored. Only 54.8% of patients who were not prescribed SGAs and did not have previous diagnoses of hypertension or hypercholesterolemia were monitored for all metabolic syndrome components compared to 92.4% of patients who had all three of these characteristics. Among patients monitored for metabolic syndrome completely, age-adjusted prevalence of the syndrome was 48.4%, with no significant difference between the three psychiatric groups.

**Conclusions:**

Only one half of patients with SMI not using SGAs or previously diagnosed with hypertension and hypercholesterolemia were completely monitored for metabolic syndrome components compared to greater than 90% of those with these characteristics. With the high prevalence of metabolic syndrome seen in this population, there appears to be a need to intensify efforts to reduce this monitoring gap.

## Introduction

Patients with serious mental illness die an average of twenty to thirty years earlier than the general population [1], with cardiovascular disease the leading cause of mortality for patients with schizophrenia [2] and bipolar disorder [3]. Metabolic syndrome is a constellation of cardiovascular risk factors including diabetes, hypertension, obesity and dyslipidemia [4], is potentially reversible and may explain the higher incidence of cardiovascular disease in patients with serious mental illness. According to the Third National Health and Nutrition Examination Survey, the age-adjusted prevalence of metabolic syndrome as defined by the US National Cholesterol Education Program Adult Treatment Panel III (NCEP ATP III) was 23.7% in the general population [5]. In contrast, a recent large study of psychiatric outpatients including patients with schizophrenia, bipolar disorder, and depression revealed a metabolic syndrome prevalence of 52% [6].

This high prevalence of metabolic syndrome seen in psychiatric patients has been partially attributed to the use of second generation antipsychotics [7], [8], especially in patients with schizophrenia. Several expert consensus guidelines have therefore called for routine monitoring of weight, lipids and glucose for patients taking second generation antipsychotics [9], [10]. Despite this, studies have shown that monitoring of metabolic syndrome components and subsequent treatment among psychiatric patients using second generation antipsychotics remains inadequate, with monitoring of different components of metabolic syndrome ranging from 0% to 70% [11], [12], [13], [14], [15], [16], [17], [18], [19], [20]. One recent study showed that monitoring failed to improve in patients taking antipsychotics even after the release of Food and Drug Administration (FDA) warnings and American Diabetic Association/American Psychiatric Association (ADA/APA) recommendations [21]. There are however no large studies that have examined monitoring of metabolic syndrome in patients with serious mental illness not using antipsychotic medications. While antipsychotic use is an important contributor to increased prevalence of metabolic syndrome among psychiatric patients, several other important factors such as physical inactivity [22], [23], poor diet [24], and cigarette use [25], [26] are also more prevalent in these patients and the presence of metabolic abnormalities has been reported in small studies of drug-naïve psychiatric patients [27], [28]. The Mount Sinai Conference has therefore suggested that all patients with schizophrenia should be considered to be at high risk for coronary heart disease and recommended routine screening for hyperlipidemia [29]. However high prevalence of metabolic syndrome is not limited to just patients with schizophrenia; patients with other serious mental illnesses such as bipolar disorder and schizoaffective disorder have also been shown to have a high prevalence of metabolic syndrome ranging from 30–70% [6], [30], [31]. This high prevalence seen in patients with different serious mental illnesses suggests that routine monitoring of the different components of metabolic syndrome in all such patients would not only be essential for early management of cardiovascular risk, but may also help guide initiation of medications such as second generation antipsychotics. Due to the aggregate nature of the syndrome, adequate monitoring for a high risk patient requires monitoring of all components of the syndrome to help guide management. However, the current literature has yet to address the present state of monitoring efforts for all the metabolic syndrome components being employed for patients with serious mental illnesses, especially those not using antipsychotic medications.

Our study therefore expands on these previous studies by examining rates of monitoring for the different components of metabolic syndrome and documents the prevalence of metabolic syndrome in patients with serious mental illness, including those not using second generation antipsychotics. For this purpose, we studied the number of patients who were not completely monitored for the five components of metabolic syndrome in a large sample of patients with a diagnosis of bipolar disorder, schizoaffective disorder or schizophrenia who received treatment at a Veterans Affairs (VA) Medical Center over a two-year period, and the prevalence of metabolic syndrome in those who were completely monitored.

## Methods

The Institutional Review Board (IRB) at the Providence VA Medical Center reviewed and approved all the procedures described in this protocol. Detailed patient information from this study was compiled by an automated search strategy using the hospital's electronic medical record system called Veterans Health Information Systems and Technology Architecture (VISTA). As a research tool, VISTA provides comprehensive and reliable patient-level clinical data across the spectrum of care and its use in research has been validated in previous trials [32], [33]. A database query of the electronic medical record was used to identify all patients with diagnoses of schizophrenia, schizoaffective disorder or bipolar disorder treated as inpatients or outpatients at the Providence VA Medical Center during calendar years 2005–2006. The study population was identified through structured search of the electronic medical records based on ICD-9 codes. This project obtained approval from the Providence VA Medical Center IRB for waiver of written informed consent as the research involves no more than minimal risk to the subjects, the waiver of consent would not adversely affect the rights and welfare of the subjects and the research could not be practicably carried out without the waiver.

The database query included only those patients who had at least one mental health visit or hospitalization during calendar years 2005–2006, so that only patients with active utilization of the VA Medical Center services would be studied. All patients were divided into three groups based on their psychiatric diagnosis – bipolar, schizoaffective or schizophrenia. Patients who had overlapping diagnoses of schizoaffective disorder with either bipolar disorder or schizophrenia were assigned to the schizoaffective disorder category [34]. Exclusion criteria included patients with no mental health visits or hospitalizations during calendar years 2005–2006 and patients who had diagnoses of both bipolar disorder and schizophrenia without a diagnosis of schizoaffective disorder.

Metabolic syndrome was defined using the NCEP ATP III guidelines [4], and consisted of meeting at least 3 out of 5 of the following criteria: (1) fasting plasma glucose ≥5.6 mmol/L (100 mg/dL) or taking anti-hyperglycemic medication, (2) triglycerides ≥1.7 mmol/L (150 mg/dL), (3) high density lipoprotein (HDL) cholesterol <1.0 mmol/L (40 mg/dL) for men and <1.3 mmol/L (50 mg/dL) for women, (4) systolic blood pressure ≥130 mm Hg and/or diastolic blood pressure ≥85 mm Hg or taking antihypertensive medication, and (5) central obesity with waist >102 cm (40 inches) for men and >88 cm (35 inches) for women. In line with previous studies [35], we used a modified ATP III criteria that substituted a body mass index (BMI)≥30 for waist circumference since data for the latter was not available.

Data collected from the medical record included demographic variables, psychiatric diagnoses, previous diagnoses of hypertension, hypercholesterolemia, diabetes, stroke, or coronary angioplasty or bypass surgery, biological variables such as BMI, systolic and diastolic blood pressures, total cholesterol, high density lipoprotein and low density lipoprotein (LDL) cholesterols, triglycerides, blood glucose, and hemoglobin A1c (HbA1c), smoking status, number of primary care provider (PCP) visits, employment and marital status, and Global Assessment of Functioning (GAF) score. Data was gathered for psychotropic medications (first and second generation antipsychotics, antidepressants, anticonvulsants, lithium, and benzodiazepines), antihypertensive medications and, anti-hyperglycemic medications. A patient was considered to have been incompletely monitored for metabolic syndrome if information required to determine the status of any one of the metabolic syndrome components, according to the modified ATP III criteria as listed above, was not available in the database query. Past diagnoses of hypertension, hypercholesterolemia and diabetes were not used to determine metabolic syndrome components, which were based on biological and medication variables from the two year study period.

### Outcomes

The main outcome of interests are the percentage of the sample population who were not monitored for all of the metabolic syndrome components and the prevalence rates of metabolic syndrome as defined by the modified ATP III criteria (BMI in place of waist circumference) among the three psychiatric groups for patients who were completely monitored for all five metabolic syndrome components.

### Statistical Procedures

After dividing patients into the three psychiatric diagnosis groups, we compared baseline characteristics, past medical history and medication prescription between the bipolar disorder, schizophrenia and schizoaffective disorder diagnostic groups. Comparisons were made using chi-square likelihood ratio tests for categorical variables or ANOVA for continuous variables. We then determined the number of patients not monitored for the five metabolic syndrome components and then compared psychiatric diagnoses, demographic variables, past medical history and psychiatric medications between patients who were incompletely monitored for metabolic syndrome and those who were completely monitored using paired Student's t-tests or likelihood ratio Chi-square tests. We then constructed a multivariate logistic regression model with incomplete monitoring as the outcome and included age, GAF, service connection, previous diagnoses of hypertension, hypercholesterolemia and diabetes and second generation antipsychotic prescription as independent variables as these are likely associated with monitoring for metabolic syndrome based on previous literature [13], [20], severity of serious mental illness [36] and likely engagement with VA providers. For patients who were monitored for all metabolic syndrome components, we compared the proportion of patients who met criteria for metabolic syndrome among the three psychiatric groups. We also calculated age-adjusted prevalence rates of metabolic syndrome using the 2000 United States census as the standard population to allow for direct comparisons with the current prevalence of metabolic syndrome in the general United States population. All statistical analyses were conducted using SAS JMP® (SAS Institute, Inc., Cary, NC). All P values were 2-sided, and a p-value of 0.05 or less was considered significant. Data was presented as mean ± standard deviation and odds ratios (OR) were reported with 95% confidence intervals (CI).

## Results

A total of 1411 patients were identified by the database query. Of these, 10 patients had diagnoses of both bipolar disorder and schizophrenia without having a diagnosis of schizoaffective disorder and were excluded from the study population. Of the 1401 included patients, 822 were placed in the bipolar disorder category, 222 in the schizophrenia category and 357 in the schizoaffective disorder category. Characteristics of the patients in these three are shown in Table 1. The mean age for the three groups was significantly different (55.7±12.8 years for the bipolar group, 60.0±11.8 years for the schizophrenia group and 53.2±10.2 years for the schizoaffective disorder group) and all three diagnostic groups were predominantly male in similar proportions. There were also significant differences among the three groups in marital status, employment status, active smoking status and GAF. Among the biological variables collected, only BMI differed significantly between the three groups, with the schizoaffective group having the highest BMI (30.3±5.8 kg/m^2^) compared to the bipolar and schizophrenia groups (29.8±5.7 kg/m^2^ and 28.9±7.3 kg/m^2^ respectively). There were significant differences among prescription of all psychiatric medication classes recorded except benzodiazepines, notably with the highest rate of second generation antipsychotic prescription occurring in the schizoaffective group (84.9%) compared to the bipolar and schizophrenia groups (49.6% and 63.1% respectively).

**Table 1:** Patient Characteristics.

	Bipolar (n = 822)	Schizophrenia (n = 222)	Schizoaffective (n = 357)	Total (n = 1401)
Age (years)*	55.7±12.8	60.0±11.8	53.2±10.2	55.7±12.3
Male	91.4%	93.6%	95.1%	92.5%
Married*	37.0%	13.2%	23.2%	29.7%
Employed*	46.9%	33.3%	34.4%	41.6%
Active Smoking*	49.7%	50.7%	57.5%	51.8%
GAF*	52.8±8.4	49.6±9.8	49.1±7.4	51.3±8.6
**Past Medical History**				
Hypertension*	46.7%	39.2%	34.7%	42.5%
Hypercholesterolemia	51.1%	45.1%	51.5%	50.3%
Diabetes	20.6%	19.8%	24.9%	21.6%
Stroke	4.0%	4.5%	3.6%	4.0%
Heart Failure	2.4%	1.8%	3.6%	2.6%
Coronary Artery Bypass Graft*	2.7%	0.5%	0.6%	1.8%
Percutaneous Transluminal Coronary Angioplasty	0.9%	0.5%	0.6%	0.7%
**Biological Variables**				
Body Mass Index (kg/m^2^)*	29.8±5.7	28.9±7.3	30.3±5.8	29.8±6.0
Systolic Blood Pressure (mm Hg)	126.0±15.7	125.0±17.3	125.1±16.5	125.6±16.1
Diastolic Blood Pressure (mm Hg)	75.5±10.3	74.1±10.1	76.0±10.7	75.4±10.4
Hemoglobin A1c (%)	6.2±0.1	6.2±1.0	6.1±1.5	6.2±1.4
Total Cholesterol (mg/dL)	190.8±45.5	184.7±41.5	188.2±44.2	189.2±44.6
High Density Lipoprotein (mg/dL)	42.7±13.3	43.8±11.4	41.1±11.8	42.4±12.7
Low Density Lipoprotein (mg/dL)	113.0±37.2	108.8±34.8	113.4±38.2	112.5±37.1
Triglycerides (mg/dL)	186.0±186.7	168.6±117.6	177.1±119.4	180.9±161.6
**Medication Prescription**				
Antidepressants*	69.8%	43.2%	64.4%	64.2%
Anticonvulsants*	64.2%	21.6%	54.1%	54.9%
Benzodiazepines	24.9%	20.7%	23.0%	23.8%
First Generation Antipsychotics*	1.2%	19.8%	11.2%	6.7%
Second Generation Antipsychotics*	49.6%	63.1%	84.9%	60.7%
Lithium*	33.2%	1.8%	24.7%	26.1%

*p<0.05 between psychiatric groups.

The number of patients in our study population who were not monitored for the five different metabolic syndrome components are listed in Table 2. Of the 1401 study subjects, 300 (21.4%) were not monitored for at least one of the five metabolic syndrome components. Additionally, 17.9% were not measured for any lipid levels (total cholesterol, triglycerides, HDL and LDL levels). A comparison of the 300 patients with incomplete monitoring (not monitored for at least one of the five metabolic syndrome components) with the other 1101 patients is shown in Table 3. There were no significant differences between these two groups in the rates of assignment to any particular psychiatric diagnosis, GAF scores, service connection and number of PCP visits over the two year period. There were significant differences, however, among the proportion of patients having previous diagnoses of hypertension, hypercholesterolemia and diabetes, with patients monitored for all metabolic syndrome components having significantly higher prevalence of these diseases(47.6%, 58.4% and 24.4% respectively) than those incompletely monitored (23.7%, 20.3% and 11.0% respectively; p<0.05 for all 3 disease states). Additionally, rates of prescription of psychiatric medications were also significantly different between the two groups, with patients completely monitored more likely to have been prescribed second generation antipsychotics and also antidepressants, anticonvulsants, and benzodiazepines.

**Table 2:** Monitoring of Metabolic Syndrome Components (n = 1401).

	Not Monitored (%)	Monitored (%)
**Metabolic Syndrome Components**		
Body Mass Index	46 (3.3)	1355 (96.7)
Hypertension	20 (1.4)	1381 (98.6)
High Density Lipoprotein	264 (18.8)	1137 (81.2)
Triglycerides	288 (20.6)	1113 (79.4)
Diabetes	104 (7.4)	1297 (92.6)
Any Metabolic Syndrome Component	300 (21.4)	1101 (78.6)
All Metabolic Syndrome Components	13 (0.9)	1388 (99.1)
**Biological Variables**		
Blood Pressure	22 (1.6)	1379 (98.4)
Hemoglobin A1c	752 (53.7)	649 (46.3)
Blood Glucose	113 (8.1)	1288 (91.9)
Total Cholesterol	251 (17.9)	1150 (82.1)
Low Density Lipoprotein	377 (26.9)	1024 (73.1)
All Lipids	251 (17.9)	1150 (82.1)

**Table 3:** Clinical and Demographic Characteristics of Patient Population by Metabolic Syndrome Monitoring Status.

	Incomplete Monitoring (n = 300)	Complete Monitoring (n = 1101)
Bipolar Disorder	61.0%	58.0%
Schizoaffective Disorder	21.7%	26.5%
Schizophrenia	17.3%	15.4%
Age (years)	54.6±15.6	56.0±11.2
Male	92.7%	92.5%
Married	29.8%	29.7%
Employed	42.0%	41.4%
Active Smoking	48.9%	52.6%
Global Assessment of Functioning	50.9±8.9	51.4±8.5
Hypertension Diagnosis*	23.7%	47.6%
Hypercholesterolemia Diagnosis*	20.3%	58.4%
Diabetes Diagnosis*	11.0%	24.4%
Stroke	3.7%	4.1%
Heart Failure	2.0%	2.8%
Coronary Artery Bypass Graft*	0.0%	0.9%
Percutaneous Transluminal Coronary Angioplasty	1.0%	2.0%
Primary Care Provider Visits	0.6±1.3	0.7±2.2
Service Connection (%)	65.9±33.5	70.9±32.7
Antidepressants*	56.0%	66.5%
Anticonvulsants*	43.3%	58.0%
Benzodiazepines*	17.3%	25.5%
Second Generation Antipsychotics*	50.3%	63.6%
Lithium	24.7%	26.4%

*p<0.05.

In the multivariate logistic regression model with incomplete monitoring as its outcome and age, GAF, service connection, previous diagnoses of hypertension, hypercholesterolemia and diabetes and second generation antipsychotic prescription as independent variables, previous diagnoses of hypertension and hypercholesterolemia and prescription of second generation antipsychotics were the only variables significantly associated with incomplete monitoring (OR = 0.73, 95% CI = 0.57 to 0.93; OR = 0.54, 95% CI = 0.42 to 0.66; and OR = 0.71, 95% CI = 0.57 to 0.88 respectively). Of patients who were not prescribed second generation antipsychotics and did not carry previous diagnoses of hypertension or hypercholesterolemia, only 54.8% were monitored for all five components of metabolic syndrome compared to 92.4% of patients who were prescribed second generation antipsychotics and carried previous diagnoses of both hypertension and hypercholesterolemia and 77.8% of patients who had at least one of these three characteristics.

For the 1101 patients who were monitored for all five components of metabolic syndrome, there were significant differences among the three psychiatric diagnostic groups in the prevalence of each of the metabolic syndrome components except triglycerides and in the number of metabolic syndrome components for which criteria were met (Table 4). However, the overall prevalence of metabolic syndrome was not significantly different among the mental illnesses: 57.1% for bipolar disorder, 50.0% for schizophrenia and 61.0% for schizoaffective disorder. Using the 2000 United States census, the overall age-adjusted prevalence rate of metabolic syndrome for the study population was 48.4%, with prevalence rates of 46.6%, 42.1% and 56.7% for bipolar disorder, schizophrenia and schizoaffective disorder respectively.

**Table 4:** Metabolic Syndrome Components for Completely Monitored Patients.

	Bipolar (n = 822)	Schizophrenia (n = 222)	Schizoaffective (n = 357)	Total (n = 1101)
Body Mass Index*	46.5%	42.9%	53.8%	47.9%
Hypertension*	78.1%	68.8%	68.8%	74.2%
High Density Lipoprotein cholesterol*	48.4%	40.6%	54.1%	48.7%
Triglycerides	49.3%	42.4%	51.4%	48.8%
Glucose intolerance*	54.0%	58.8%	63.7%	57.3%
Metabolic Syndrome	57.1%	50.0%	61.0%	57.0%
Number of Criteria Met*	2.8±1.4	2.5±1.5	2.9±1.4	2.8±1.4
Age-Adjusted Metabolic Syndrome	46.6%	42.1%	56.7%	48.4%

*p<0.05 between psychiatric groups.

## Discussion

With cardiovascular disease being the leading cause of mortality among patients with serious mental illness, monitoring for metabolic syndrome, a major modifiable risk factor for cardiovascular mortality, is paramount for early detection and management. Since previous studies have focused solely on psychiatric patients using second generation antipsychotics, our study investigated current monitoring rates of metabolic syndrome in a large number of patients with serious mental illness, including patients not using antipsychotic medications. Our study showed that one fifth of the study population was not monitored for at least one of the five metabolic syndrome components over a two year period. However, only half of the patients who were not prescribed second generation antipsychotics and were not diagnosed with hypertension or hypercholesterolemia previously were monitored for all metabolic syndrome components compared to 92.4% of patients who had all three of these characteristics. Among the patients who were completely monitored for metabolic syndrome, the overall age adjusted prevalence of metabolic syndrome was 48.4%, grossly double that of the general United States population [5].

The high prevalence of metabolic syndrome seen in patients with serious mental illness, especially those taking second generation antipsychotics, has led to the development of several expert guidelines recommending routine metabolic monitoring in patients using antipsychotics [9], [10] and those with schizophrenia [29]. Despite this, monitoring and treatment of various components of metabolic syndrome for patients using antipsychotic agents remains inadequate [11], [12], [13], [14], [15], [16], [17], [18], [19], [20], [21]. While our study showed that roughly 80% of patients with serious mental illness were completely monitored for all components of metabolic syndrome, monitoring was much lower in patients not prescribed second generation antipsychotics and without a previous history of hypertension or hypercholesterolemia. These results are similar to one previous study which saw higher rates of serum glucose and lipid testing in patients using second generation antipsychotics who carried previous diagnoses of diabetes or dyslipidemia [13]. The rates of monitoring of the different metabolic syndrome components in our study are higher than two previous studies examining monitoring of all metabolic syndrome components in patients using second generation antipsychotics which ranged from 0% to 40% [15], [16]. The overall higher monitoring rates in our study may be partially explained by the organizational pursuit of more integrated health care, especially between primary care and mental health services, at a VA Medical Center compared to non-VA settings [37] where most previous studies have been conducted. Specifically, co-location of mental health and non-mental health services in one setting, as occurs at a VA Medical Center, is associated with improved health monitoring of mental health patients [38]. Additionally the use of performance measures in the Veterans Health Administration to hold managers and clinicians accountable for appropriate management and monitoring of chronic diseases and conditions such as weight and exercise counseling for obese patients, periodic monitoring of blood pressure, lipids and glycemic control for patients with diabetes, arranging early follow-up after discharge for patients with multiple anti-psychotic medications, tobacco counseling and annual depression screening, among others [39], is likely an important contributor to the higher monitoring rates seen in our study. It is also likely that the patients in our study had a higher comorbidity burden warranting increased monitoring of various health parameters, including metabolic syndrome components, irrespective of their mental health diagnoses as has been seen previously in comparisons of veteran and non-veteran patients [40], [41]. One previous study of veterans with bipolar disorder using second generation antipsychotics revealed that roughly 50% had been monitored for cholesterol and triglyceride levels and 70% for serum glucose levels [18], which are rates closer to those seen in our study.

Among patients in our study who were completely monitored for metabolic syndrome, our results confirmed the high prevalence rates seen in previous studies, with prevalence more than double that of the general population [5]. Several studies have shown metabolic syndrome to be more prevalent among patients with schizophrenia than the general population, with prevalence among some recent American and Canadian studies ranging from 41% to 52% [6], [31], [42], [43], [44], [45]. Although smaller in number, other studies have shown increased prevalence of metabolic syndrome among patients with bipolar disorder and schizoaffective disorder as well, with a prevalence of 30 to 70% for bipolar disorder [6], [31], [46], [47], [48] and 42.4% to 67% for schizoaffective disorder [30], [31]. However our study is among only a few studies with large sample sizes which have examined metabolic syndrome components among patients with serious mental illness. Our study differs from other large studies such as the work by Rejas et al. (2007) [49], de Hert et al. (2009) [50] and Shi et al. (2009) [51] in that it includes patients with serious medical illnesses other than just schizophrenia and also patients not using antipsychotic medications. Another large study of 10,084 psychiatric outpatients with schizophrenia, bipolar disorder, and depression was performed by Correll et al. [6]. But unlike the Correll et al. study which included only motivated and likely healthier individuals willing to participate in a voluntary metabolic health fair, our study is a cross-sectional analysis of all in- and out-patients coming to the VA Medical Center over a two year period and likely includes a broader spectrum of patients with major mental illnesses irrespective of their disease severity. Although not statistically significant, our study did show that patients with schizoaffective and bipolar disorders trended towards higher prevalence of metabolic syndrome than those with schizophrenia. These findings are similar to a recent study of Australians with severe mental illness [31], which found prevalence of metabolic syndrome to be greatest among patients with bipolar or schizoaffective disorder (both 67%), followed by schizophrenia (51%) and may be suggestive of a link between affective symptoms and risk for metabolic syndrome. Patients with affective symptoms (bipolar disorder and schizoaffective disorder) are known to have Hypothalamus-Pituitary-Adrenal (HPA) axis dysregulation [52] which may result in hypercortisolism and contribute to weight gain, hypertension and insulin resistance, a milieu favorable for the development of metabolic syndrome. Additionally, use of certain anti-depressant medications, which are more likely to be used in patients with affective symptoms compared to those with schizophrenia alone, has been associated with metabolic abnormalities [53].

According to the ADA/APA consensus statement, patients using second generation antipsychotics should routinely have their BMI, waist circumference, blood pressure, fasting plasma glucose and fasting lipid profile monitored [9]. While the use of second generation antipsychotics has been associated with metabolic syndrome in several studies [7], [8], psychiatric patients may also be at risk for metabolic syndrome even in the absence of medication use. Studies have revealed higher rates of impaired glucose tolerance [27] and visceral fat distribution [28] in drug-naïve patients with schizophrenia compared to healthy controls. Psychiatric patients are more likely to have reduced access to health care which combined with socioeconomic factors limits the identification and management of medical conditions such as metabolic syndrome [54]. Additionally, the unaffected first degree relatives of patients with schizophrenia have higher rates of type 2 diabetes mellitus [55] suggesting a possible genetic association between schizophrenia and metabolic abnormalities, with the 5,10-methylenetetraydrofolate reductase (MTHFR) gene a possible candidate for such a link [56], [57]. Due to the strong association between schizophrenia and cardiovascular disease, the Mount Sinai Conference recommended that patients with schizophrenia should be considered to be at high risk for coronary heart disease [29] which would qualify them for more frequent monitoring. Our study, however, suggests that in addition to following these existing guidelines, providers should consider screening all patients with serious mental illnesses for metabolic syndrome due to the high prevalence rates of this condition observed in patients with bipolar and schizoaffective disorders along with those with schizophrenia.

Our study has certain limitations. Due to the cross-sectional nature of this study, we are unable to draw any conclusions regarding the causality of any associations seen in the study and the population described in this study consists exclusively of veterans who are predominantly male, white and over 50 years old, which may not reflect the demographic characteristics of these disorders outside the VA setting. However, this design allows us to use a relatively large sample size to determine current monitoring efforts in patients with different serious mental illnesses, including those not using second generation antipsychotics. Secondly, we modified the NCEP ATP III criteria (substituted a BMI≥30 for the waist circumference criterion) which is likely to have somewhat altered the proportion of people in our sample categorized as having metabolic syndrome. However, this modification is consistent with previous studies of its kind [35], and reflects current knowledge. Additionally, since providers are less likely to measure waist circumference compared to BMI, our estimation of monitoring rates of psychiatric patients is likely to be a more conservative one than reality. Our study while looking at the rates of monitoring for metabolic syndrome in the VA, did not account for the possibility that patients may have been monitored for different metabolic syndrome components at non-VA facilities. However, the likelihood of this is low since we selected patients who were actively being treated at the institution. Additionally, there were no significant differences in the average service connection and the number of PCP visits between patients who were completely monitored and those who were not, suggesting that both groups likely had similar degrees of engagement with their providers, either at the VA or non-VA institutions. However, we did not specifically measure potential provider based predictors affecting monitoring of patients with serious mental illness for metabolic syndrome. Due to the nature of the database query, the mental health diagnosis and measurements of the different metabolic syndrome criteria were likely not made during the same clinical visits. Additionally, it is not possible to discern whether or not DSM-IV criteria were strictly followed in determining psychiatric diagnoses or whether or not these were done in the setting of structured interviews.

Our study expands on existing literature by examining monitoring of all five metabolic syndrome components among patients with different psychiatric disorders including those not using second generation antipsychotics. Our results show that about 80% of patients with serious mental illness were monitored for all metabolic syndrome components over a two year period. However, monitoring appears to be significantly lower among patients not prescribed second generation antipsychotics or without previous diagnoses of hypertension and hypercholesterolemia. These results, coupled with the high prevalence of metabolic syndrome – more than double that of the general population – seen in our study, suggest a need to intensify monitoring of metabolic syndrome among all patients with serious mental illness, including for those not using second generation antipsychotics.

### Disclaimer

The views expressed in this article are those of the authors and do not necessarily reflect the position or policy of the Department of Veterans Affairs.
